# Morphological and Microstructural Alterations of the Articular Cartilage and Bones during Treadmill Exercises with Different Additional Weight-Bearing Levels

**DOI:** 10.1155/2017/8696921

**Published:** 2017-07-11

**Authors:** Jiazi Gao, Juan Fang, He Gong, Bingzhao Gao

**Affiliations:** ^1^State Key Laboratory of Automotive Simulation and Control, Jilin University, Changchun, China; ^2^Department of Engineering Mechanics, Nanling Campus, Jilin University, Changchun, China

## Abstract

The aim of this study was to investigate the morphological and microstructural alterations of the articular cartilage and bones during treadmill exercises with different exercise intensities. Sixty 5-week-old female rats were randomly divided into 10 groups: five additional weight-bearing groups (WBx) and five additional weight-bearing with treadmill exercise groups (EBx), which were subjected to additional weight bearing of *x*% (*x* = 0, 5, 12, 19, and 26) of the corresponding body weight of each rat for 15 min/day. After 8 weeks of experiment, the rats were humanely sacrificed and their bilateral intact knee joints were harvested. Morphological analysis of the cartilages and microcomputed tomography evaluation of bones were subsequently performed. Results showed that increased additional weight bearing may lead to cartilage damage. No significant difference was observed among the subchondral cortical thicknesses of the groups. The microstructure of subchondral trabecular bone of 12% and 19% additional weight-bearing groups was significantly improved; however, the WB26 and EB26 groups showed low bone mineral density and bone volume fraction as well as high structure model index. In conclusion, effects of treadmill exercise on joints may be associated with different additional weight-bearing levels, and exercise intensities during joint growth and maturation should be selected reasonably.

## 1. Introduction

Osteoporosis and its consequential fragility fractures are among the leading causes of morbidity, thereby causing considerable and growing social and economic burden [1–3]. Bone mass is considered to be a key determinant of fracture risk [1], and peak bone mass is suggested to be the most important factor in the development of osteoporosis [1]. Maximizing bone mass during childhood and adolescence may contribute to the reduction of fracture risk in the elderly [1]. In addition, physical activities may be an important contributor to the increase in bone mass [4–10]. A study on 4-week-old female Sprague-Dawley rats showed that 8 and 12 weeks of exercise (treadmill running at 24 m/min, 1 hr per day, 5 days a week) substantially increased the mineral apposition and bone formation rates in the proximal and distal tibial metaphyses and increased the cancellous bone volume in the proximal tibial metaphyses [4]. In another study that subjected 4-week-old male Wistar rats to treadmill running exercises (30 m/min, 1 hr per day, 5 days a week for 10 weeks), the increase in bone strength induced by exercise was mediated by the changes in the trabecular bone microarchitecture, as well as in the density and cortical geometry [10].

Moreover, increasing evidence showed that the effects of physical activities on the bones of growing rats persisted after exercise cessation [8, 9, 11, 12]. For example, 4-week-old male Wistar rats that underwent treadmill training for 10 weeks (35 m/min, +5-degree inclination, 1 hr per day, 5 days a week) had greater bone mineral content and longer bones than the rats in the control group, and the increased bone mass due to training was retained after the cessation of training [11]. Furthermore, the forearm axial compression loading for 3 days a week for 7 weeks on 5-week-old Sprague-Dawley rats significantly improved bone mineral content, areal density, and strength. These improvements remained until the rats were two years of age [12]. These findings suggest that exercise at a young age provides lifelong benefits to bone structure and strength. These benefits are expected to increase the peak bone mass and reduce the risk of fracture due to aging in the later years; in addition, the prepubertal period is also suggested to be the most effective stage for physical activity interventions [8, 13].

In prepubertal rats, although weight-bearing physical activities play an important role in the accrual of bone mass and maintenance of bone quality, the effects of exercise on other tissues, such as articular cartilage and subchondral bone, should not be disregarded, especially when the bones and cartilages are growing at a fast rate. The biomechanical environment can initiate degenerative changes on immature articular cartilage during joint growth and maturation [14]. Excessive running exercises (15 km within 3 weeks or 30 km within 6 weeks) in 13- to 14-week-old male Wistar rats induced knee osteoarthritis [15]. Furthermore, 16- to 18-week-old Wistar rats forced to run 30 km on a treadmill platform for 6 weeks also suffered from osteoarthritis [16]. In another study, an in vivo tibial loading model was used to assess the influence of mechanical load on articular cartilage and bone; the results showed that in vivo cyclic compression of 4.5 and 9.0 N peak loads via the knee joint (1200 cycles, 4 Hz, 5 days a week) caused cartilage degeneration and subchondral bone changes in 10- and 26-week-old mice [17].

Overall, these studies verified the following assumptions: (1) physical activities can improve bone quality and prevent osteoporosis due to aging, (2) physical activities at a young age may enhance bone quality and the prolong exercise-induced benefits until old age, and (3) improper (e.g., excessive) physical activities may increase the risk of osteoarthritis to the joints.

Most studies on physical activities at a young age focused on bone tissues and paid little attention to the joints. Therefore, in this study, we focused on the influence of physical activities on the articular cartilages and bones (including subchondral bone and proximal tibial trabecular bone) of growing rats. In the present study, growing rats were subjected to continuous treadmill running. A morphological analysis of the cartilages and microcomputed tomography (micro-CT) evaluation of the bones were then performed. The peak strain during exercise can be increased by adding weights [18], and thus, exercise intensity was regulated by adjusting the addition of weights during exercise in this study.

## 2. Materials and Methods

### 2.1. Animals

All the experimental procedures were approved by the Ethics Committee of The First Hospital of Jilin University (number 2013-145).

A week before the experiment, 85 female Wistar rats, aged 4 weeks, were purchased and brought to the laboratory so that they can acclimate to the new environment. All the rats were housed in cages (6 rats in each cage) under local vivarium conditions (temperature 24 ± 2°C and 12 hr on/off light cycle). Free cage movement was allowed. The rats were provided with standard pelleted chow diet and water in the entire experimental period, and no dietary adjustments were made.

### 2.2. Experimental Design

According to previous studies, 12 m/min was considered to be a “gentle” running (walking) speed on account of the onset of blood lactate accumulation [19], which was confirmed in the present study. On the last day of acclimation, 25 rats were selected and randomly divided into either the sedentary group (*n* = 5) or the exercise group (*n* = 20). The rats in the exercise group were subjected to treadmill exercise at speeds of 8, 10, 12, and 14 m/min (*n* = 5 for each speed) for 15 min; they were then sacrificed immediately after exercise. Blood samples from the rats were collected via the abdominal aorta before death and then centrifuged at 3000 rpm for 15 min. The serums were separated, and the blood lactate concentrations were determined.

No significant blood lactate accumulation was observed until the running speed increased to 14 m/min (the median value of the blood lactate concentration was 4.5 mmol/L). Therefore, 12 m/min (the median value of the blood lactate concentration was 2.5 mmol/L, which was significantly lower than that at 14 m/min, *P* < 0.05) showed only a slight blood lactate accumulation and thus can be regarded as the highest threshold level of gentle running. As such, 12 m/min was selected in the present study.

**Figure 1:**
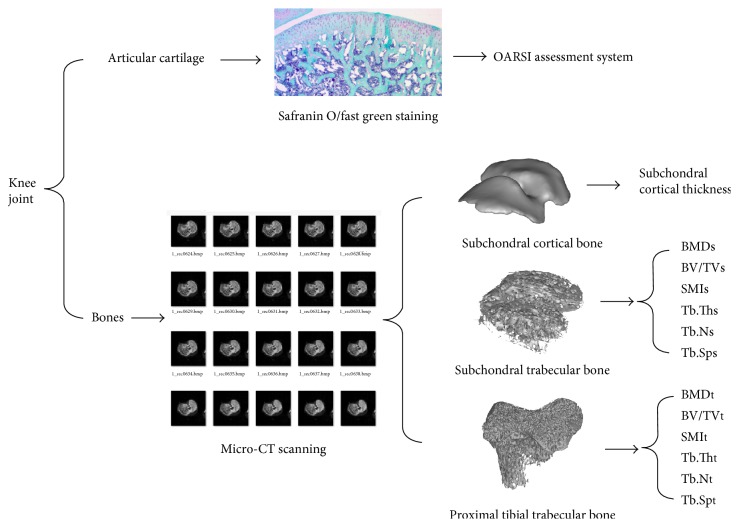
Assessment of morphological and microstructural alterations of the articular cartilage and bones.

Subsequently, the other 60 rats were randomized into 10 groups with 6 rats each (Table 1). Additional weight bearing was carried out by allowing the rats to carry a backpack filled with leaden strips [18]. All the rats in the exercise groups were subjected to running exercise on a rodent treadmill platform. The speed of the treadmill and the weight-bearing of each rat were gradually increased during the first 14 days. That is, the running speed was increased from 8 m/min, with 3% increment, to 12 m/min. The weight of the backpack was increased from 0% of the individual weight until the targeted additional weight in increments of 7% of the experimental requisite weight. All the experimental groups were maintained for an additional 8 weeks (7 days per week) as an experimental period. The additional loads in the backpack were adjusted in accordance with the change in the body weights of the rats per week. After the experiment, the rats were humanely killed under general anesthesia using sodium pentobarbital. Bilateral intact knee joints were harvested and fixed in 10% formalin for 24 hr. Morphological analysis of the articular cartilage and micro-CT evaluation of the bones were then performed (Figure 1()).

### 2.3. Morphological Analysis on the Articular Cartilage

After tissue fixation, the right knee joints were decalcified in ethylenediaminetetraacetic acid for 2 weeks, and then dehydrated in a series of alcohol baths, and finally embedded in paraffin. Serial sagittal sections (6 *μ*m thick) were obtained using a rotary microtome. Safranin O/fast green staining was performed to assess the articular cartilage morphology. Articular cartilage degeneration was assessed in tibial plateau using the OARSI osteoarthritis cartilage histopathology assessment system [20].

### 2.4. Microstructural Analysis on Bones

After the tissues were fixed, the left knee joints were scanned by a high-resolution micro-CT scanner (Skyscan 1076, Skyscan, Belgium) set at 18 *μ*m resolution, 70 kV, 142 *μ*A, and an Al 1.0 mm filter. Quantitative microstructural analysis of bones was then performed.

Subchondral bone includes subchondral cortical bone and subchondral trabecular bone. Thus, the subchondral cortical thickness and microstructural parameters of the subchondral trabecular bone, including bone mineral density (BMD_S_), bone volume fraction (BV/TV_S_), structure model index (SMI_S_), trabecular thickness (Tb.Th_S_), trabecular number (Tb.N_S_), and trabecular separation (Tb.Sp_S_), were calculated. In addition, microstructural parameters of the proximal tibial trabecular bone under growth plate (BMD_t_, BV/TV_t_, SMI_t_, Tb.Th_t_, Tb.N_t_, and Tb.Sp_t_) were also quantified to compare with those of the subchondral trabecular bone.

### 2.5. Statistical Analysis

Given that the data were nonnormally distributed, nonparametric tests of significance were performed. Two independent variables (exercise and additional weight bearing) with different levels (exercise or not; additional weight bearing of 0%, 5%, 12%, 19%, and 26%) were analyzed using the nonparametric two-way analysis of variance (Scheirer-Ray-Hare test). The Mann–Whitney *U* test was then used to compare the morphological and microstructural parameters between every two groups. A *P* value < 0.05 was considered statistically significant [21, 22].

## 3. Results

### 3.1. Evaluation of Articular Cartilage Degeneration in the Tibial Plateau

**Figure 2:**
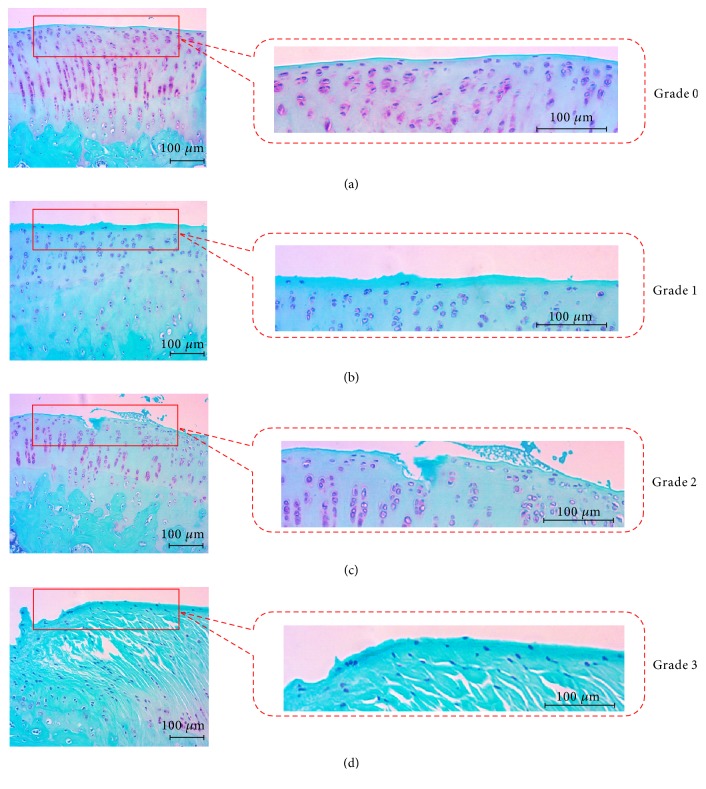
OARSI assessment of articular cartilage ((a) grade 0: cartilage surface is smooth; (b) grade 1: surface intact with uneven articular surface is observed; (c) grade 2: discontinuity of surface is destroyed; and (d) grade 3: cartilage degeneration is observed with obvious cracks extension).

The results of the OARSI assessment are shown in Table 2. No change in the cartilages of WB0, EB0, WB5, and EB5 were observed. WB12 and EB12 showed minimal surface discontinuities (Figure 2(a)). WB19, EB19, and WB26 had same sample distribution in terms of their OARSI grades (Figures 2(b) and 2(c)). However, EB26 showed obvious cartilage damage. In this group, only 3 of 6 samples were in grade 0, and 1 of 6 samples was in grades 1, 2, and 3. Visualized Safranin O staining loss and cartilage thinning were observed in EB26 (Figure 2(d)).

### 3.2. Microstructural Evaluation of Subchondral Bone

The microstructural parameters of the subchondral bone were analyzed, and the results are shown in Table 3.

No significant difference in subchondral cortical thickness was observed among groups.

Statistical analysis results show that exercise or the interaction between exercise and weight-bearing level had no significant effect on any parameter of the subchondral trabecular bone (Scheirer-Ray-Hare test: exercise or not, *P* > 0.05; interaction between exercise and weight-bearing level, *P* > 0.05). Subchondral trabecular bone changes in the BMD_S_, BV/TV_S_, SMI_S_, Tb.Th_S_, and Tb.Sp_S_ were mainly dependent on the weight-bearing level (Scheirer-Ray-Hare test: weight-bearing level, *P* < 0.05). High BMD_S_ was observed in the 12% additional weight-bearing groups (WB12 and EB12), which had significantly higher BMD_S_ than EB0. For BV/TV_S_, both 12% and 19% additional weight-bearing groups and EB5 showed higher values than WB0 (*P* < 0.05). EB12 had the highest BV/TV_S_ (higher than WB0 and EB0, *P* < 0.05). Although the 12% and 19% additional weight-bearing groups showed low SMI_S_, a significant difference was only observed from EB12 and WB0 (*P* < 0.05). No difference among the groups was observed with respect to Tb.N_S_. The 12% and 19% additional weight-bearing groups showed higher Tb.Th_S_ than EB0 (*P* < 0.05). EB5, WB12, EB12, and EB19 showed significantly lower values than EB0 in terms of Tb.Sp_S_. Among the groups, EB12 had the lowest Tb.Sp_S_ (significantly different from WB0 and EB0, *P* < 0.05).

### 3.3. Microstructural Evaluation of Proximal Tibial Trabecular Bone

The microstructural parameters of the proximal tibial trabecular bone are shown in Table 4.

Results of proximal tibial trabecular bone show the similar trend with the subchondral trabecular bone. That is, exercise or the interaction between exercise and weight-bearing level had no significant effect on any parameter of the proximal tibial trabecular bone (Scheirer-Ray-Hare test: exercise or not, *P* > 0.05; interaction between exercise and weight-bearing level, *P* > 0.05). Microstructural parameters (BMD_t_, BV/TV_t_, SMI_t_, Tb.Th_t_, and Tb.Sp_t_) were mainly dependent on the weight-bearing level (Scheirer-Ray-Hare test: weight-bearing level, *P* < 0.05). The 0% additional weight-bearing groups (WB0 and EB0) showed lower BMD_t_ than other groups, with the highest value observed in EB12. For other microstructural parameters, EB12 showed the highest BV/TV_t_ (significantly higher than WB0 and EB0, *P* < 0.05), Tb.N_t_, and Tb.Th_t_ (significantly higher than EB0, *P* < 0.05) and the lowest SMI_t_ and Tb.Sp_t_ (significantly lower than EB0, *P* < 0.05).

## 4. Discussion

The influence of different exercise intensities on the articular cartilage and bones of growing rats was investigated in this study. The rats were subjected to continuous treadmill running with different additional weight bearings. The cartilages and bones were then subjected to morphological analysis and micro-CT evaluation, respectively.

With regard to the running speed, 12 m/min corresponded to a moderate level by evaluating blood lactate accumulation in the present study. Apparent blood lactate concentration was observed when the running speed increased to 14 m/min, which means the intensity of the exercise has exceeded our expectations. Besides, lower speeds (such as 8 m/min or 10 m/min) were still rejected to avoid confusion with voluntary exercise. Accordingly, the speed of 12 m/min was selected in the current study.

Although treadmill running could enhance bone quality [4, 10, 11], the influence of running on cartilage should not be ignored. Moderate mechanical loading could maintain the integrity of the cartilage and prevent the progression of cartilage-subchondral bone lesions [23, 24]. However, overuse of joints may result in cartilage degradation [15, 16, 25]. In this study, articular cartilage degeneration was clearly observed in the 26% additional weight-bearing groups (WB26 and EB26). The 12% and 19% additional weight-bearing groups showed different grades of cartilage changes. The results of the assessment of cartilage in the tibial plateau indicated that the increase in additional weight bearing exacerbated cartilage damage. This effect may be related to the immature cartilage in growing rats and thus imply the importance of selecting beneficial exercise intensity during the growing period of cartilage and bones.

Subchondral bone, which plays an important role in load distribution and support in joints, is closely associated with osteoarthritis and thus is a tissue of great interest [26–34]. Whether the onset of osteoarthritis occurs in the bone or articular cartilage remains controversial. Several studies suggested that changes in the subchondral bone occur following the degeneration of the cartilage [26, 27]. However, many researchers speculated that changes in the bone occur simultaneously with cartilage degradation or even before cartilage degradation [28, 29]. Mechanical factors play a significant role in the physiologic imbalance of osteoarthritis. The health and integrity of the overlying articular cartilage depend on the mechanical properties of its bony bed [30]. The mechanical effects of loading not only influence the bone mass but also alter the subchondral bone [34]. Although no significant change in the subchondral cortical bone was observed in the current study, the subchondral trabecular bone exhibited substantial changes. The results of this study showed that the 12% and 19% additional weight-bearing groups considerably improved the microstructure of the subchondral trabecular bone, but the positive results were not observed in the 26% additional weight-bearing groups. In addition, although no significant difference was found between WB26 and EB26, they showed low BMD_S_ and BV/TV_S_, as well as high SMI_S_, thus indicating that the 26% additional weight bearing failed to improve bone quality. Therefore, increased exercise intensity cannot improve further exercise-induced positive effects, especially in growing joints, which may be associated with the ability of bone adaption especially that of growing bones. Though this study provided a proper additional weight-bearing level (19% additional weight bearing) for the growing bones and joints, a further study is still needed to explore the reasonable exercise intensities more precisely.

Microstructural parameters of the proximal tibial trabecular bone under the growth plate showed similar changes with the subchondral trabecular bone. However, the changes of BMD_t_ seemed more sensitive; that is, not only 12% and 19% additional weight-bearing groups but also 5% additional weight-bearing groups showed significant higher values than 0% additional weight-bearing groups. Though those results may not be helpful to understand the load transduction with regard to bones and joints under treadmill exercises with different additional weight-bearings levels in growing individuals, they still suggested that the proper exercise intensities could improve bone quality for the growing individuals.

The current results should be considered in light of several limitations. First, no additional weight bearing heavier than 26% of the individual body weight was used in this study because the adaptability of the rats was considered. Thus, the effects of additional weight bearing heavier than 26% on articular cartilage and subchondral bone were not discussed. However, articular cartilage degeneration was clearly observed in the 26% additional weight-bearing groups, which means it is unnecessary to enlarge the range of an additional weight-bearing value. Second, the models used in this study lacked exact evaluation of the strains produced by different exercise intensities in the joints. The relationship between mechanical stimulation (strain) and changes in the joint (articular cartilage and subchondral bone) was not discussed, which should be improved in the future studies to conduct a quantitative and comprehensive research. Third, the results obtained in the paper were based on female rats, which means that the conclusion might not be applicable to the male rats. The differences between genders should be investigated deeply in the future to extend the conclusion of this study. Although restricted by these limitations, the influence of physical activity (treadmill running) on articular cartilage and subchondral bone were investigated, and the results may provide insights into the means of enhancing bone quality.

In conclusion, different exercise intensities did not considerably affect the subchondral cortical thickness but influenced the articular cartilage structure, as well as the microstructural parameters of subchondral trabecular bone and proximal tibial trabecular bone. The results of this study suggested that the exercise intensities during joint growth and maturation should be selected reasonably to improve bone quality and avoid the risk of osteoarthritis due to aging.

## Figures and Tables

**Table 1:** Groups' information and experimental design.

Groups	Number	Experimental design
WBx (*x* = 0, 5, 12, 19, 26)	6 × 5 groups	Additional weight-bearing groups with additional weight bearing at *x*% of the individual body weight, backpack for 15 min/day with no weight bearing or treadmill exercise at other times.
EBx (*x* = 0, 5, 12, 19, 26)	6 × 5 groups	Exercise groups combined with additional weight bearing at *x*% of the individual body weight, treadmill exercise with backpack for 15 min/day with no weight bearing or treadmill exercise at other times.

**Table 2:** OARSI grades of cartilage (the numbers of cartilages in each grade).

Grade	WB0	EB0	WB5	EB5	WB12	EB12	WB19	EB19	WB26	EB26
Grade 0	6	6	6	6	5	4	4	4	4	3
Grade 1	0	0	0	0	1	2	1	1	1	1
Grade 2	0	0	0	0	0	0	1	1	1	1
Grade 3	0	0	0	0	0	0	0	0	0	1

**Table 3:** Microstructural parameters of subchondral bone.

Groups	Subchondral cortical thickness (mm)	Subchondral trabecular bone					
		BMD_S_ (g/cm^3^)	BV/TV_S_ (%)	SMI_S_	Tb.N_S_ (1/mm)	Tb.Th_S_ (mm)	Tb.Sp_S_ (mm)
WB0	0.1840	0.6145	36.6019	1.1143	2.6330	0.1365	0.2727
EB0	0.1849	0.6106	35.9493	1.1867	2.6300	0.1378	0.2844
WB5	0.2017	0.6147	41.1165	1.0059	2.7910	0.1395	0.2841
EB5	0.1978	0.6131	43.2750^∗^	1.0251	2.9930	0.1427	0.2662^#^
WB12	0.1840	0.6231^#^	41.4677^∗^	0.9902	2.7920	0.1457^#^	0.2611^#^
EB12	0.1893	0.6260^#^	44.0822^∗^^,#^	0.9188^∗^	2.8620	0.1534^#^	0.2452^∗^^,#^
WB19	0.2045	0.6190	41.4688^∗^	0.9976	2.9990	0.1488^#^	0.2585
EB19	0.1860	0.6185	41.6078^∗^	1.0281	2.8290	0.1506^#^	0.2588^#^
WB26	0.1952	0.6065	39.3952	1.2407	2.8670	0.1367	0.2764
EB26	0.1971	0.5997	39.8096	1.3481	2.6430	0.1346	0.2895

^∗^Significantly different from the WB0 group; *P* < 0.05. ^#^Significantly different from the EB0 group; *P* < 0.05.

**Table 4:** Microstructural parameters of the proximal tibial trabecular bone.

Groups	BMD_t_ (g/cm^3^)	BV/TV_t_ (%)	SMI_t_	Tb.N_t_ (1/mm)	Tb.Th_t_ (mm)	Tb.Sp_t_ (mm)
WB0	0.5583	25.3438	1.8431	2.2250	116.9828	242.2194
EB0	0.5528	23.6330	1.7450	2.0890	111.1648	264.1477
WB5	0.5767^#^	30.1409	1.6501	2.2050	130.5708	265.4428
EB5	0.5713^#^	29.1304	1.7560	2.1260	127.7959	233.0518
WB12	0.5747^∗^^,#^	30.9775	1.8210	1.8530	131.9947	262.4064
EB12	0.5950^∗^^,#^	44.9278^∗^^,#^	0.9834^#^	2.4910	163.0242^#^	228.0384
WB19	0.5688^#^	32.2411	1.6022	2.0080	128.8975	212.6581
EB19	0.5740^∗^^,#^	38.3036	1.1095	2.0800	143.4085	225.0497
WB26	0.5645	21.2810	1.9553	1.8500	119.9064	287.3870
EB26	0.5603	21.7358	2.1625	1.8030	122.8960	272.5016

^∗^Significantly different from the WB0 group; *P* < 0.05. ^#^Significantly different from the EB0 group; *P* < 0.05.
